# Comparative Study on the Behavior of Reinforced Concrete Beam Retrofitted with CFRP Strengthening Techniques

**DOI:** 10.3390/polym14194024

**Published:** 2022-09-26

**Authors:** Aditya Kumar Tiwary, Sandeep Singh, Raman Kumar, Kamal Sharma, Jasgurpreet Singh Chohan, Shubham Sharma, Jujhar Singh, Jatinder Kumar, Ahmed Farouk Deifalla

**Affiliations:** 1 Department of Civil Engineering, Chandigarh University, Mohali 140413, India; 2 University Centre of Research and Development, Mechanical Engineering Department, Chandigarh University, Mohali 140413, India; 3 Institute of Engineering and Technology, GLA University, Mathura 281406, India; 4 Department of Mechanical Engineering, IK Gujral Punjab Technical University, Main Campus, Kapurthala 144603, India; 5 Department of Mechanical Engineer, St. Soldier Institute of Engineer and Technology, Punjab 144001, India; 6 Structural Engineering and Construction Management Department, Future University in Egypt, New Cairo 11835, Egypt

**Keywords:** beam, CFRP, ultimate load, deflection, retrofitting, failure mechanism

## Abstract

Lateral reinforcement has a significant impact on the strength and ductility of concrete. Extra confinement is provided in this project by carbon fiber reinforced polymer (CFRP) sheets wrapped around the outside of reinforced concrete (RC) beams. To determine the failure criteria and maximum load-carrying capacity of beams, numerous specimens were cast and tested in a flexural testing machine. This paper presents the results of an experimental investigation of functionally damaged reinforced concrete beams repaired in flexure with CFRP sheets. The most essential variable in this study is the CFRP sheet scheme, and seven different strengthening schemes (B1 to B7) were explored in the experimental program. In conclusion, the findings of the study showed that flexural retrofitting of reinforced concrete beams with CFRP sheets is functionally effective, with restored strength and stiffness values roughly equivalent to or greater than those of the control beam (CB1). The efficiency of the flexural retrofitting mechanism appears to vary depending on the layout of the CFRP sheet. Steel rupture and concrete crushing were shown to be the most common failure modes in the investigation, causing CFRP sheets to break in retrofitted beams.

## 1. Introduction

The need for rehabilitation in the civil construction sector nowadays stems from structural deterioration/aging, the adaptation of old structures to new design standards, design errors, accidental overloading, and a change in the structure’s operational needs. The goal of rehabilitation, repair, and strengthening is to create cost-effective, long-lasting structures [1]. The magnitude of earthquake motion has greatly increased. Their frequency has also increased; earthquakes typically last only a few seconds, but the devastation they produce is always disastrous. Even a little earthquake might result in considerable property damage. In the event of a major earthquake, the entire structure may collapse, resulting in the loss of human life and significant property devastation, which has an indirect impact on the owner’s economy. Disposal of debris after an earthquake is another issue that can be difficult and expensive. Even in the event of a moderate earthquake, the structure may not totally collapse, but fissures may appear, which may become the cause of a significant failure in future earthquakes. The decision now is whether to demolish the entire house and rebuild it, which is a highly expensive operation, or to rehabilitate the entire structure. There are numerous methods for rehabilitation, and depending on the intended output, any method can be chosen, such as beam strengthening, steel jacketing, carbon fiber retrofitting, and concrete jacketing. This is significant because beams are critical structural elements for sustaining loads and finding effective strengthening solutions is critical for ensuring the structures’ safety. The use of fiber-reinforced plastic (FRP) has expanded significantly in recent years. In comparison to external bracing or steel jacketing, it is more reliable for seismic retrofitting of RC structures. FRP has a flatter and imperceptibly attractive surface compared to steel plates because FRP is composite materials made of a polymer matrix reinforced with fibers that manifest numerous outstanding mechanical characteristics such as superior strength stiffness to weight ratio, resistance to corrosion, tensile strength, durability, lightweight, ease of handling, lower maintenance costs, and faster installation time [2]. CFRP and glass fiber reinforced polymers (GFRP) are the main fibers for reinforcing the material. Vinyl ester, epoxy, and polyester thermosetting plastics, as well as phenol–formaldehyde resins, are the most often used polymers. The aerospace, automotive, marine, and construction industries all use FRP composite materials [3].

To reinforce the weak member, FRP material is employed. FRP material comes in a variety of shapes and sizes, including bars, sheets, and laminates. Externally, these materials use adhesive to adhere to the defective member and offer strength. These materials outperform other materials because they have high mechanical strength, are lightweight, and are simple to work with. Garden and Hollaway (1998) [4] demonstrated that the attributes of FRP materials are superior to steel, particularly in terms of tensile strength, and that these traits may be achieved throughout a wide temperature range.

The critical performance of reinforced concrete beams retrofitted with carbon fiber reinforced polymer was examined by Rahimi et al. [5]. The research was carried out on 2–3 m long RC beams that had been covered with CFRP sheets. Internal primary steel and external bonding materials were the factors. In order to compare steel jacketing to other external steel procedures, steel plate bonding has also been considered. Aside from laboratory work, the theoretical study was completed to ensure that the practice is correct. As a result, a good comparison was made between laboratory work and non-linear software work. The necessity for structural retrofitting comes in two situations: (i) when the structures must be used for situations where the load is greater than the design load, and (ii) when existing structures must be improved. This review study delves into the materials and procedures for upgrading RC beams in buildings using fiber-reinforced polymer (FRP).

Large skill beams were regarded as the destruction of beams by Sheikh S.A. et al. [6]. The foundation walls continue to wreak havoc. The elements were retrofitted with reinforcing materials such as carbon and glass fiber reinforced polymer (CFRP and GFRP) sheets, and their failure was tested. In order to acquire precise results, control beams were also taken into consideration for comparison. Retrofitting of reinforced concrete Haunched Beams (RCHBs) using Carbon FRP (CFRP) and Glass FRP (GFRP) strips was also covered in this research. Furthermore, the behavior of FRP laminates in the retrofitting of RC beams subjected to high temperatures was investigated. The effectiveness of various types of FRP materials and processes was also considered.

RC beams are strengthened and upgraded for a number of reasons [7,8,9,10,11,12,13]. On one of the three faces of the original cross-section, the RC Jacket is one of the most important approaches for strengthening RC structural components [8,14,15,16,17,18]. The use of GIWWM as an outer reinforcement and incorporated within a larger section is generally thought to be a promising technique for strengthening, repairing, rehabilitating, and even retrofitting reinforced concrete sections. This method not only enhances the load capacity of reinforced beams, but it also improves their ductility [19,20,21,22].

Galvanized iron welded wire mesh (GIWWM) is a type of construction material made comprised of electrically welded rods that are woven together to form a continuous, uniformly dispersed mesh. GIWWM has various advantages [23,24,25] due to its relative ease of placing, bending, and handling, as well as its high strength-to-weight ratio. In terms of lightweight, durability, and fire resistance, it performs other reinforcing functions. Several researchers recently examined the bond strength between older and younger concrete in enhancing the bond at the contact surface and attaining full composite action capacity [26,27,28]. The concrete substrate is exposed to a wide range of damage and deterioration variables in real life, which can be divided into two groups [29,30,31,32,33]. The first is immediate damage, which provides natural disasters, conflicts, and unforeseen consequences; the second is progressive damage, which includes exploitation, negligence, and hazardous external conditions [34,35,36,37] such as carbonation, sulfate assault, chloride attack, and alkali–silica interaction. Some of these element’s aid in the bonding of the newly cast concrete to the damaged concrete substrate [38]. On a number of composite RC components, experiments and analytical verification have been conducted [39,40,41]. The flexural behavior of composite RC beams reinforced with SCC or other materials has been examined in several research [42,43,44].

The repaired jacketed beams have been tested after being strengthened externally with light-gauge steel wiring mesh embedded in 2.0 cm thick grout, which improved their deformation capacity and loading strength [45]. Zhang et al. [46] examined the flexural behavior of reinforced RC T-beams with self-compacting concrete (SCC) jacketing at the tension zone, as well as the performance of these RC beams under multiple sustaining loads. The strengthening approach significantly improved the flexural behavior and stiffness of strengthened beams, according to the results of the testing. By employing a high-temperature chemical vapor deposition (CVD) furnace, Duong et al. [47] present an overview of the floating catalyst approach for fabricating continuous macroscopic fibers and films from CNT super fibers. This procedure allows for the one-step production of vast quantities of aligned carbon nanotube fiber (CNT) assemblies with precise control over their shape. From a green perspective, the procedure is appealing because of the benefits it offers with regard to energy, time consumption, expenses, and waste materials. The electrical and mechanical properties of CNT fibers were investigated by Duong et al. [48] after being subjected to a variety of post-treatment. The post-treated CNT fibers enhanced mechanical and electrical performance is comparable to that of many commercial high-strength fibers, suggesting their great potential in a wide range of applications, including structural reinforcements, supercapacitors, flexible heaters, medical devices, and lightweight electric cable.

Wan et al. [49] examined the effects of water on the binding between carbon fiber reinforced polymer (CFRP) and concrete before, during, and after the CFRP cure. The interfacial energy release rate, G, of the CFRP-concrete bond is calculated using modified double cantilever beam (MDCB) specimens. The test results show that the bond quality is greatly reduced when water is present during the CFRP application, and the majority of the failures that arise are adhesive failures at the primer/concrete interface. Even though the bond capacity is slightly higher when a specially designed primer is used, undesirable failure still occurs. High-quality CFRP installations that were exposed to water after the epoxy had dried for only a short time between 3 to 8 weeks showed that the binding between CFRP and concrete was weakened.

Jiang and Wu [50] looked into how the load eccentricity affected the axial strength of short concrete columns that were FRP-confined. According to the test results, FRP confinement can result in less strength loss than unconfined concrete specimens. The strength improvement brought on by FRP confinement for square concrete specimens rises with increasing load eccentricity. For FRP-confined circular concrete specimens, the confinement efficiency is reduced as the load eccentricity increases.

Taking into account model uncertainty, Zhang et al. [51] investigated a comprehensive reliability-based analytical methodology for FRP-to-concrete bonded joints. The bond strength models for FRP-to-concrete bonded joints were calibrated by defining a model factor, and then eight of the most popular models were utilized to determine the calibration factors. All eight model parameters might be characterized as normally distributed random variables with a lognormal distribution by employing this method of characterization. Reliability research proved the value of having calibrated models share similar uncertainty.

The flexural performance of CFRP strengthened beams has not been extensively studied, as evidenced by the literature cited above, and further research is needed to fully comprehend the behavior of reinforced concrete beams. The peculiarity of this study is that it uses an extensive experimental test program to demonstrate the performance and usability of CFRP sheets in reinforced cement concrete as a strengthening approach for RC beam members. As a result, the flexural load capacity and failure patterns of these reinforced specimens have been tested experimentally. The study was also expanded to look into the behavior of beams with different retrofitting techniques, such as steel jacketing and concrete jacketing. The specimens’ maximum load-carrying capacity, load Vs deformation, and stress Vs strain were all studied. With this goal in mind, the objectives were as follows: (1) to use CFRP sheets to test the flexural capability of strengthened sles; (2) to investigate the failure mode, crack width, and crack pattern of pre-cracked reinforced concrete beams retrofitted with CFRP sheets at various positions and lengths, and (3) to compare the mode of failure of control reinforced concrete beams and reinforced concrete beams retrofitted with CFRP sheets at various positions and lengths.

## 2. Specimen Details and Material Properties

The goal of this research is to reinforce and restore damaged and weak structures. Different strengthening schemes must also be investigated, as no examination into the essence of the CFRP sheet scheme into the performance of preloaded beams restored with CFRP for flexural strengthening has been conducted. The flexural performance of RC beams retrofitted with CFRP sheets was investigated in this study. Experiments on full-size beams were carried out in the lab to achieve this. The CFRP sheet schemes are the study’s main variables. M30 grade of concrete has been cast as per guidelines of IS 10262: 2019 in the Concrete Technology Laboratory, Chandigarh University, Mohali, India. The experimental work uses M30 grade concrete and tests beams using a two-point loading technique, with the major focus on beam flexural behavior. The cross-section of all beams was the same, as were the flexural and shear reinforcement characteristics. One beam was designated as the control beam (CB1). The remaining seven beams were retrofitted with various CFRP sheeting techniques. There was only one loading strategy employed.

Seven beams, B1, B2, B3, B4, B5, B6, and B7, were pressured until flexural cracks appeared, and then reinforced with CFRP sheet coating, which have been procured from the Vision Infra Solutions, Mumbai, India Mart, India. To retrofit the beams, an FRP system was used, with only one layer of CFRP being coated on all retrofitted beams which have been procured from the Vision Infra Solutions, Mumbai, India Mart, India. The strengthened beams were then placed in the universal testing machine (UTM) (Mechatronic Control System of Capacity 0–1000 kN, Structural Engineering Laboratory, Chandigarh University, Mohali, India) to be loaded again until failure occurred, and the results were compared to the controlled beam (CB1).

### 2.1. Specimen Details

**Figure 1:**
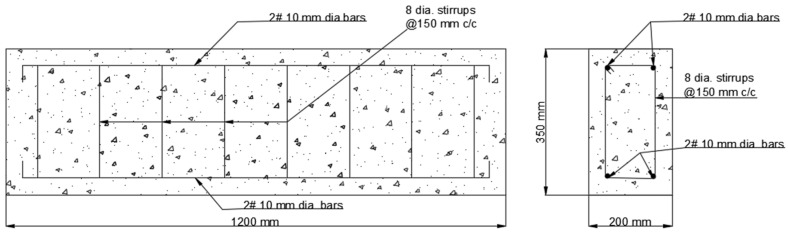
Reinforcement detail of beam.

**Figure 2:**
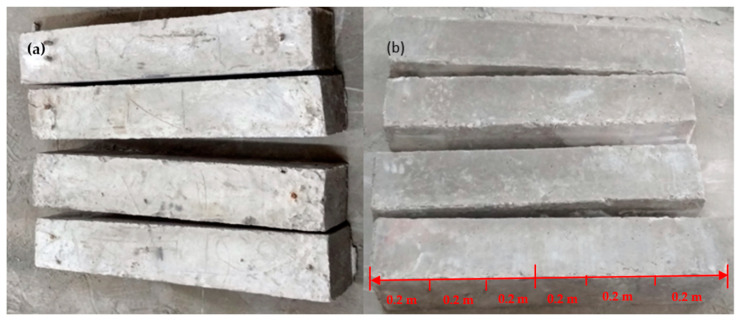
(**a**,**b**) Prepared beam specimens without CFRP sheets.

**Figure 3:**
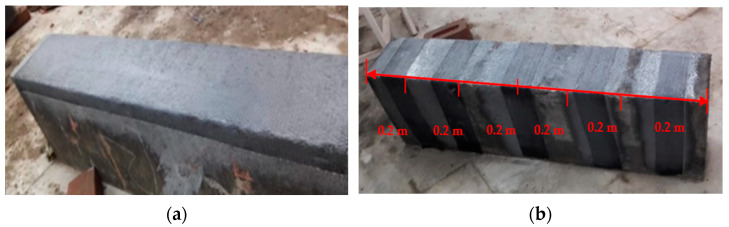
(**a**,**b**) Prepared beam specimens with CFRP sheets.

At the time of testing, all of the beams had identical size, flexural, and shear reinforcement, (Structural Engineering Laboratory, Chandigarh University, Mohali, India) and were 28 days old. All beams had a rectangular cross-section with a length of 1200 mm, a width of 200 mm, and a depth of 350 mm. Two 10 mm bars were considered for flexural fortification at the soffit, and the top reinforcement of each beam was 10 mm, with stirrups of 8 mm spaced 150 mm c/c throughout the beam length as shown in Figure 1. Figure 2a,b and Figure 3a,b depict the prepared beam with and without CFRP sheets for experimental testing. In this study, the concrete grade was M30, and the steel was Fe500.

### 2.2. Material Used for Casting of Beam

**Figure 4:**
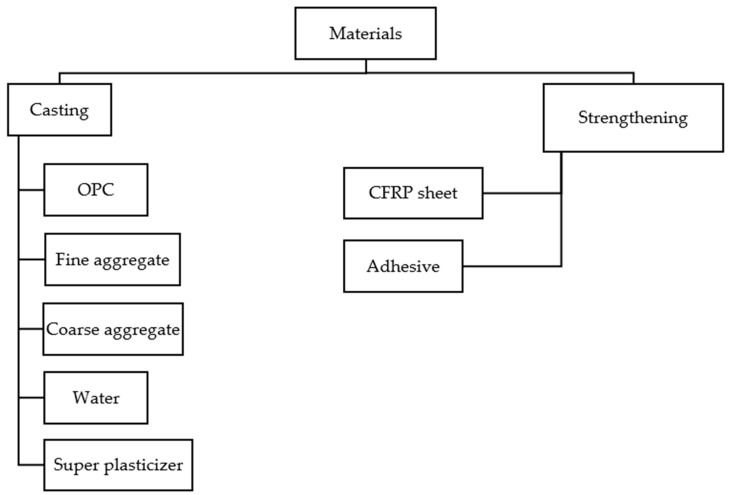
Material used for casting of beams.

The various materials used for casting beams are discussed in Figure 4.

#### 2.2.1. Ordinary Portland Cement (OPC)

Limestone and other powdered raw ingredients such as calcareous, argillaceous, and gypsum are used to make this type of cement. The entire testing was done with OPC 43 grade (Ultra-Tech Cement, Ultra-Tech Industry, Chandigarh, India) in this inquiry. Table 1 lists the OPC’s qualities according to IS 8112: 2013. The cement should be stored in a stack and out of the way of moisture. Fineness, specific gravity, consistency, setting time, and compressive strength are among the several types of tests conducted on cement.

#### 2.2.2. Aggregates 

The most commonly utilized aggregates are crushed gravel, crushed rock, and sand, which are all readily available. The primary function of fine aggregates is to aid in the delivery of working and consistent results in the combination. Aggregates (locally available, Chandigarh, India) make up 60% to 75% of concrete volume. The aggregates are then divided into two groups.

Coarse Aggregates: Coarse aggregates are defined as those that are reserved on an IS sieve size of 4.75 mm. Coarse aggregates include materials such as natural gravel (locally available, Chandigarh, India) and crushed stone (locally available, Chandigarh, India). The average aggregate size used in concrete is 10–20 mm; however, self-compacting concrete uses sizes up to 40 mm. The aggregates’ grade is almost as significant as their quality. The workability, homogeneity, and finishing quality of concrete are all affected by aggregate gradation [52,53,54]. Locally available coarse aggregates with diameters of 20 mm and 10 mm were recycled in this project near Chandigarh. The aggregates were first washed, then submerged in water for 24 h to remove dust and other organic material, then cleaned and dried to a saturated surface dry condition. IS: 383-1970 standards were used to assess the aggregates [55,56,57]. Table 2 lists the specific gravity as well as a variety of other parameters. Table 3 shows the sieve analysis of the coarse aggregate.

**Table 2:** Properties of coarse aggregates.

Property	Value
Specific gravity	2.71
Shape	Angular
Fineness Modulus	2.25
Color	Grey
Maximum size	20

**Table 3:** Sieve analysis of course aggregate.

IS Sieve Size (mm)	Weight of Aggregate Retained				% of Total Weight Retained	Cumulative % Retained	% Age Passing
	I	II	III	Avg.			
20	23	35	61	39.7	3.8	3.6	96.1
16	113	63	89	86.3	8.6	13.6	86.3
12.5	271	179	280	250.8	23.9	25.9	64.4
10	352	389	329	349.6	34.7	73.3	28.1
4.75	239	323	239	269.8	27.9	100.1	0.7
PAN	9	11	3	7.0	0.7

(1) $$\mathrm{Fineness}\;\mathrm{Modulus}=\frac{\sum^{\;}\left(\mathrm{Cumulative}\;\%\;\mathrm{Retained}\right)}{100}=2.33$$

Fine Aggregates: Fine aggregates are those that pass through an IS sieve with a size of 4.75 mm. Natural sand was commonly used in India. The natural sand has the advantage of having rounded or cubical particles with a smooth surface texture. The grade of the sand differs from one location to the next. Because it is cubical, rounded, and smooth textured, it is easy to deal with. Zone III sand (locally available, Chandigarh, India) was used in this experiment, which satisfies the code criteria (IS: 383-1970) [58,59,60]. The sand was fine and the shading was brown. Table 4 lists the fine aggregates’ physical parameters as well as the results of the sieve analysis. The fine aggregate sieve analysis is presented in Table 5.

**Table 4:** Physical properties of fine aggregates.

Property	Value
Specific Gravity	2.70
Fineness	2.79
Water Absorption	0.6%

**Table 5:** Sieve analysis of fine aggregate.

IS Sieve Size (mm)	Weight of Aggregate Retained				% of Total Weight Retained	Cumulative % Retained	% Age Passing
	I	II	III	Avg.			
10 mm	-	-	-	-	-	-	-
4.75 mm	40	30	38	36	3.6	3.6	96.4
2.36 mm	31	28	28	29	2.9	6.5	93.5
1.18 mm	52	40	42	44.7	4.5	10.9	89.0
600 *μ*	82	74	72	76	7.6	18.6	81.4
300 *μ*	318	238	290	282	28.2	46.8	53.2
150 *μ*	438	510	464	470.7	47.1	93.8	6.2
75 *μ*	36	68	56	53.3	5.3	99.2	0.8
PAN	3	12	10	8.3	0.8	-	-

(2) $$\mathrm{Finess}\;\mathrm{Modulus}=\frac{\sum^{\;}\left(\mathrm{Cumulative}\;\%\;\mathrm{Retained}\right)}{100}=2.78$$

#### 2.2.3. Superplasticizer

Sikaplast 4202 NS (Vision Infra Solutions, Mumbai, India Mart, India) was utilized as a superplasticizer to reduce the amount of water in a mix design while increasing concrete strength. This superplasticizer was obtained from SIKA and meets IS 9103-1999 requirements [61,62,63]. The HRWR, or high-rate water reducer (locally available, Chandigarh, India), is a superplasticizer that makes concrete workable with very little water. The doses recommended by the corporate expert should be between 0.5 and 2 percent by weight of cement. Table 6 shows the chemical and physical parameters of the superplasticizer that was utilized.

#### 2.2.4. CFRP Sheet

**Figure 5:**
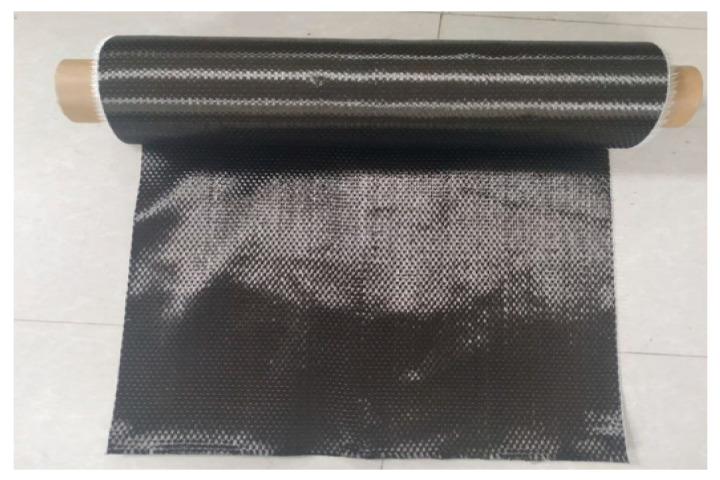
CFRP sheets (Sika wrap-230C).

A carbon fiber reinforced polymer (CFRP) sheet was employed for the strengthening and restoration of RC beams, as seen in Figure 5. A single sheet of CFRP is wrapped around the concrete specimen. In this investigation, CFRP sheets from SIKAWRAP-230C were employed which have been procured from the Vision Infra Solutions, Mumbai, India Mart, India, which come in rolls with a width of 500 mm, a length of 50 mm, and a cross-sectional area of 25 m^2^. These sheets are frequently utilized in all types of concrete structures to improve the structure’s strength and load-carrying capacity. The properties of CFRP sheets were determined in the laboratory. The tensile modulus (*E_f_*) of CFRP was 230 GPa and strain (ε*_Rupture_*) was 1.7%. The density of CFRP sheet was 1.82 g/cm^3^ and tensile strength (*F_f_*) was 4000 MPa of 0.13 mm thick CFRP sheets. 

#### 2.2.5. Sikadur 330 IN

Sikadur 330 IN epoxy adhesive (Sika India Pvt. Ltd., Navi Mumbai, India) was used to wrap the CFRP sheet around the concrete specimen. Sikadur 330 IN is a thixotropy epoxy-based impregnating resin/adhesive that comes in two pieces.

## 3. Experimental Program

The entire experimentation performed in the structural Engineering laboratory, Chandigarh University, Mohali, India.

### 3.1. Loading on Controlled Beam

**Figure 6:**
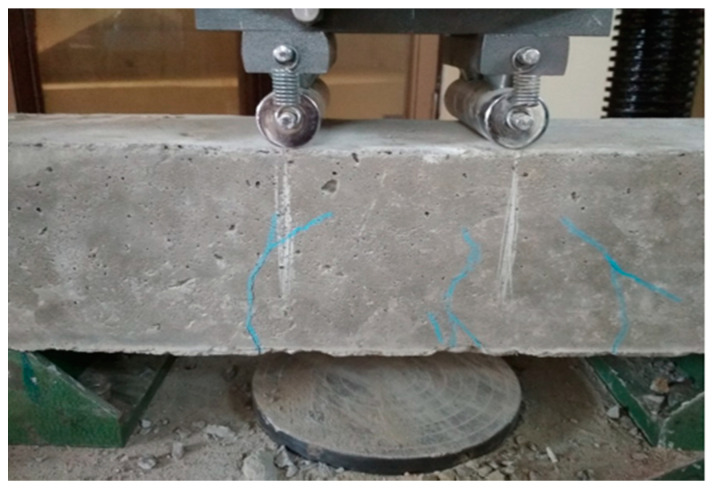
Loading setup on beam.

**Figure 7:**
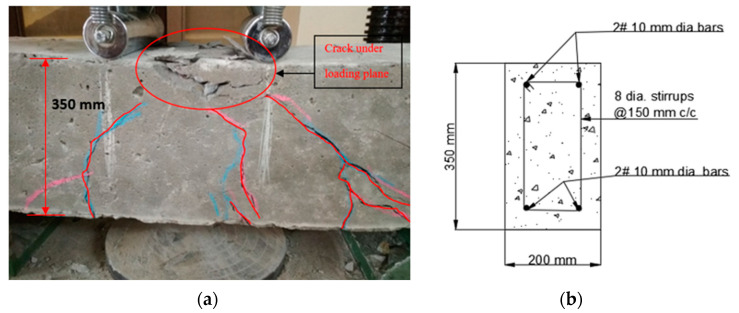
(**a**,**b**) Failure of controlled beam.

Before retrofitting, the beams B1 to B7, as well as the control beam CB1, were preloaded to simulate impairment, as indicated in Figure 6. The beams were initially loaded until the first crack appeared around P = 160 kN, after which the load was released. This preload was equivalent to about 43% of the unstrengthen beam’s maximum loading capacity. The highest deflection was found to be around 11.4 mm as can be seen in Figure 7a,b.

### 3.2. Retrofitting of Beams

**Figure 8:**
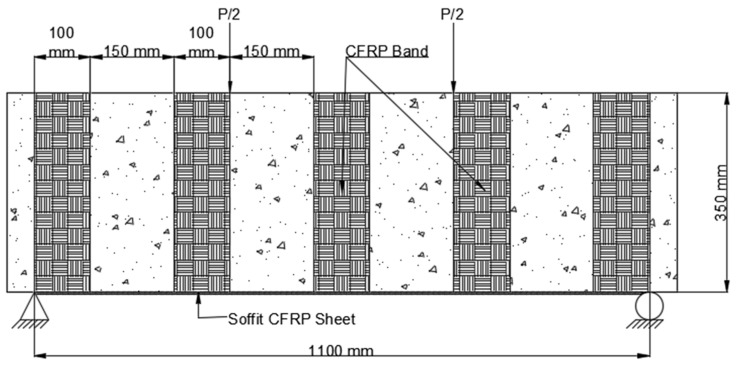
Placement of CFRP on beam Scheme 1 (B1).

**Figure 9:**
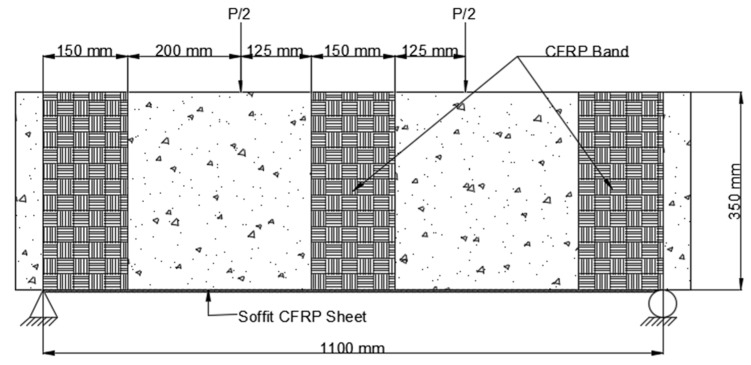
Placement of CFRP on beam Scheme 2 (B2).

**Figure 10:**
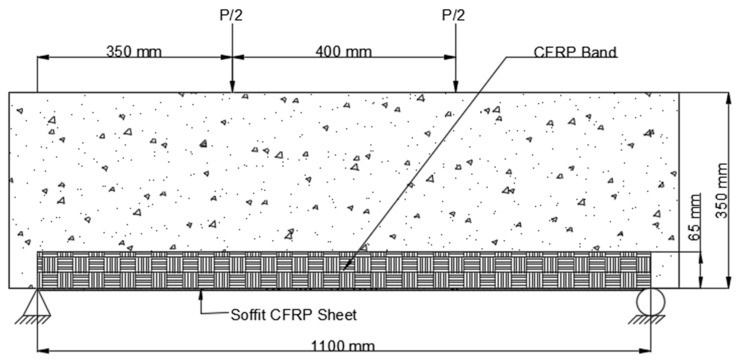
Placement of CFRP on beam Scheme 3 (B3).

**Figure 11:**
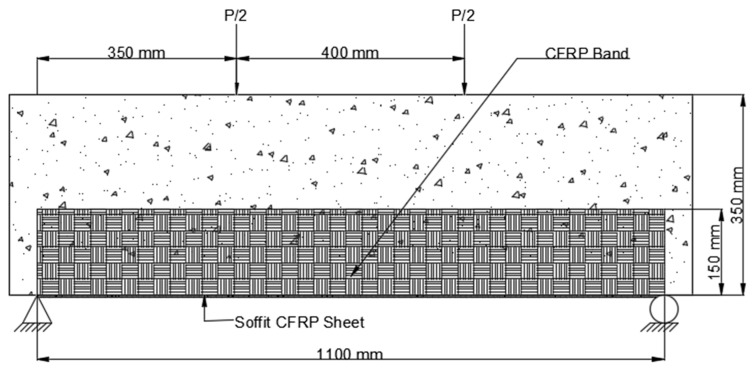
Placement of CFRP on beam Scheme 4 (B4).

**Figure 12:**
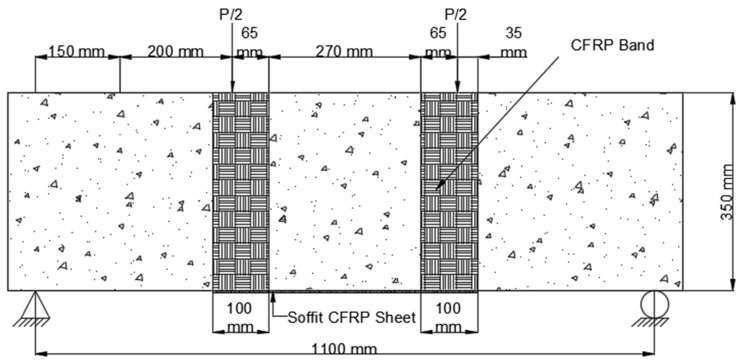
Placement of CFRP on beam Scheme 5 (B5).

**Figure 13:**
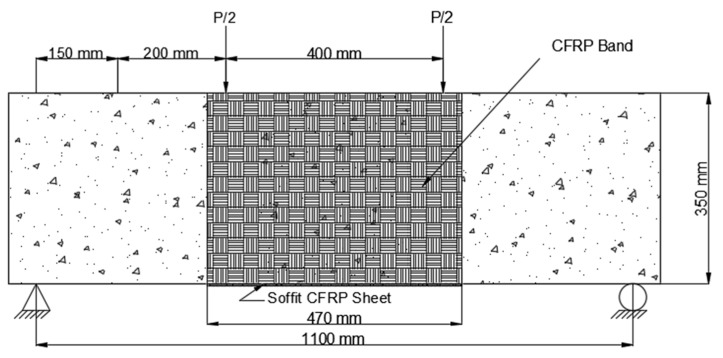
Placement of CFRP on beam Scheme 6 (B6).

**Figure 14:**
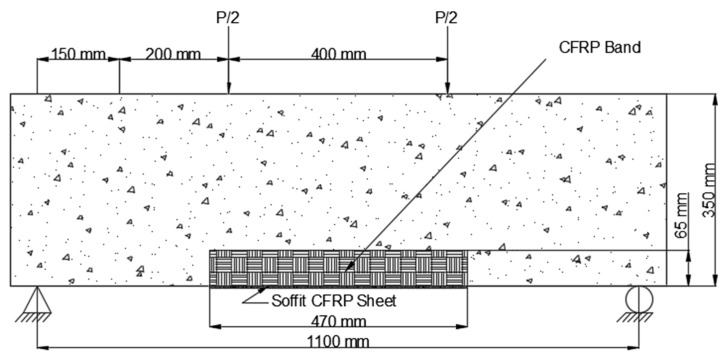
Placement of CFRP on beam Scheme 7 (B7).

As shown in Figure 8, Figure 9, Figure 10, Figure 11, Figure 12, Figure 13 and Figure 14, preloaded beams were evacuated from the Universal Testing Machine and turned for retrofitting. For retrofitting damaged beams, seven distinct solutions were considered. There were two series of these seven different schemes: A and B. The four strengthening schemes in Series A were as follows:

1. As depicted in Figure 8, the U-straps were located every 150 mm and wrapped the beam’s bottom surface, as well as both sides and faces, to a height of 350 mm in Scheme 1 (B1). The U-straps were 100 mm in width. The U-shaped CFRP sheets were applied to reinforce the beams in both shear and flexural modes, as well as to demonstrate a flexural failure model.

2. Two U-straps were mounted at the end of the tension face CFRP sheet and one in the center of the beam in Scheme 2 (B2), which was similar to Scheme 1. They encased the soffit and extended it on both sides of 350 mm in height. The U-straps had a width of 150 mm. Figure 9 shows how U-straps were used to secure the CFRP flexural face sheet and prevent it from debonding.

3. The CFRP sheet used in Scheme 3 (B3) was 1100 mm long and 200 mm wide, with fibers arranged parallel to the axis of the beam. This CFRP sheet was encased across the side faces of the beam to a height of 65 mm above the tension face, as shown in Figure 10.

4. Scheme 4 (B4) was similar to Scheme 2, with the exception that the CFRP sheet was enclosed across the side faces of the beam to a height of 150 mm above the tension face, as shown in Figure 11.

In three separate schemes, the CFRP sheets in Series B were extended to 42% of the beam span. The goal of this study was to see if preloaded beams in the hogging moment zone may improve their cracking resistance and flexural capacity. The three strengthening schemes in Series B were as follows:

1. The CFRP sheet was implemented in the constant moment area in Scheme 5 (B5). Two CFRP U-straps with fibers aligned in the direction of the beam’s longitudinal axis, which was bounded by the soffit, were mounted under the load point. The CFRP sheet was 470 mm long and 200 mm broad, and the fibers aligned in a direction corresponding to the longitudinal axis of the beam, which was confined to the beam’s soffit (i.e., oblique direction). Thoroughly wrapped the beam’s bottom surface and covered both sides to a width of 100 mm and a height of 350 mm, as shown in Figure 12.

2. The constant moment zone, which was 470 mm long and 250 mm wide, was also applied to Scheme 6. (B6). As indicated in Figure 13, the CFRP sheet was wrapped across the beam side to a height of 350 mm above the tension face of the beam.

3. Scheme 7 (B7) was equivalent to Scheme 3, except the CFRP sheet was 370 mm long and placed at the constant moment zone (see Figure 14).

## 4. Results and Discussions

### 4.1. Load-Deflection Behavior

**Figure 15:**
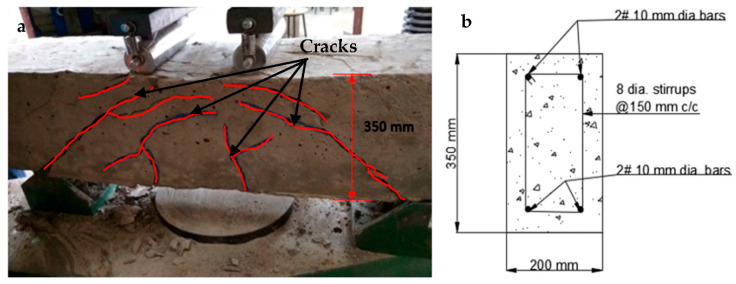
(**a**,**b**) Formation of the crack in controlled beam (CB1).

**Figure 16:**
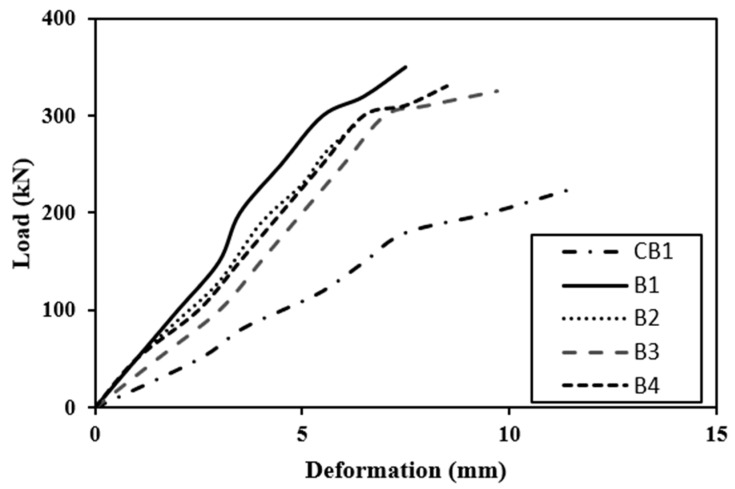
Load deflection behavior of strengthened and control beam.

**Figure 17:**
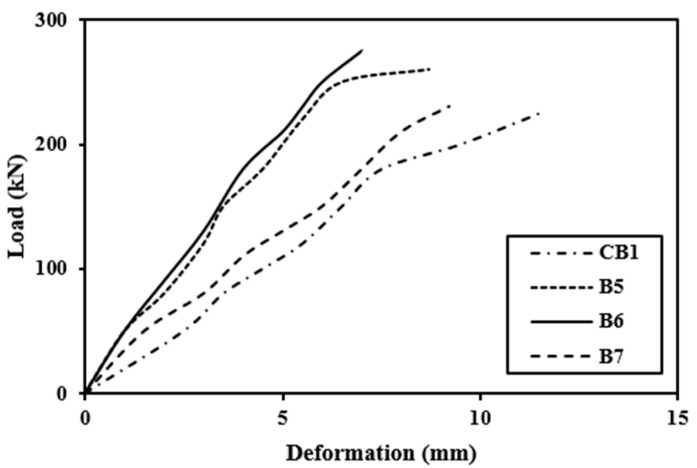
Load deflection behavior of strengthened and control beam.

The crack formation with the application of load is shown in Figure 15 and the load-deflection behavior of the beam is shown in Figure 16 and Figure 17. A dial gauge was positioned at the mid-span of the test beam to acquire current data, and a hydraulic system was used to provide load. The mid-span deflection data for a 20 mm deflection was recorded. The fundamental reason for this is that none of the specimens failed in attaining that value. All modified beams were inspected under a Universal Testing Machine until failure occurred after 5 days of applying the CFRP sheets. The research was conducted using the same identical configuration as the control beam section testing and the results are identical with the prior studies [64,65,66].

#### 4.1.1. Controlled Beam (CB1)

Control beams have a ductile nature and may withstand a lot of deflection before finally failing. At a load of 160 kN, flexural cracks formed in the positive moment area with a crack diameter of less than 1 mm as can be seen in Figure 15a,b. The linearity of concrete cracking is indicated by the deflection curve for load mid-span. As the load raised, the cracks developed and extended into the compression zone of the beam. A maximum load of 224 kN was reported. As the testing continued to the maximum load, no cracks were observed, but bending cracks spread in the positive moment zone [67,68,69]. The beam failed due to normal steel yielding prior to concrete crushing, with the concrete crushing happening precisely around the point load [1,2,3].

#### 4.1.2. Strengthened Beams

Figure 16 and Figure 17 illustrate that the stiffness of beams at minor loads is essentially identical. Control beam rigidity drops dramatically after a load of around 135 kN (roughly cracking phase) due to cracking. Because the CFRP sheet layer prevents cracks from forming and expanding, retrofitted beams have a reduced drop-in stiffness. Series A reinforces the beam by bonding the full tension face with the partial wrapping of the side faces or by connecting the tension face bonding with U-shaped CFRP sheets. This is most probably due to the fact that side face wrapping of a U-shaped CFRP sheet or beam, in conjunction with whole tension face bonding, provided anchoring and hence was very effective in the cracking zone. The beams are less flexible because only the hogging moment region has been bonded because the tension face CFRP sheet with the partial wrapping of the side faces of the beam as in B3 and B4 is in Series B. The stiffness of a fully U-wrapped maximum moment area is significantly higher than that of the other two schemes in Series B. It is essential to mention that if the control beam is loaded until it fractures, then unloaded, then loaded again, the stiffness will decline attributable to the second time beam damage [4,70,71].

The load-deflection curves reveal that the strengthening technique significantly enhanced the beam’s ultimate strength as compared with the control beam. The ultimate load in Scheme 1 of Series A was 350 kN, a 56% growth above the control beam. The final strength of Scheme 2 was 295 kN, which indicated a 30% enhancement over the control beam. Scheme 3’s ultimate load was 328 kN, which was 46% higher than the control beam. The final load for Scheme 4 was 330 kN, a 47% growth over the previous load. The ultimate load in Scheme 5 of Series B was 265 kN, an increment of 18% over the control beam. Scheme 6 had a 275 kN ultimate strength, which was 23% higher than the control beam. Scheme 7 yielded a maximum load of 230 kN, eliminating the structural failure caused by preloading.

### 4.2. Ductility Factor

**Figure 18:**
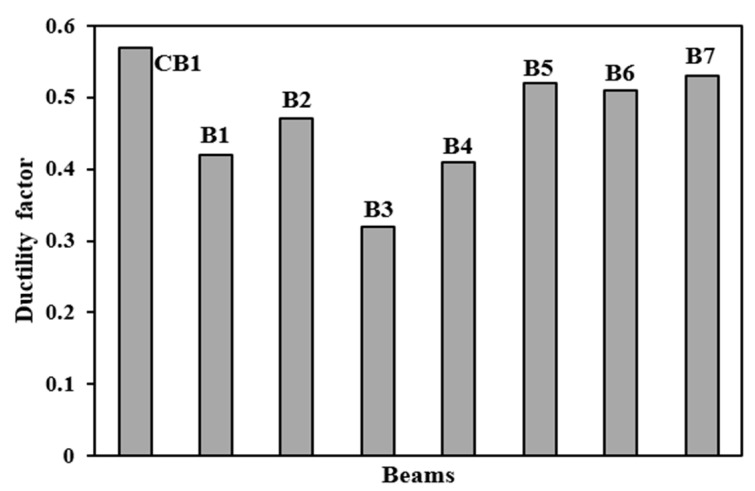
Ductility factors of control and retrofitted beams.

The ductility factors of beams were measured in order to estimate the ductile qualities. The ductility factor, as shown in Figure 18, is the ratio of RC beam deflection at cracking load to deflection at maximum load. The larger the ductility parameters, the more the beams are wrapped at the hogging moment area [5,9]. In comparison to the other designs, Scheme 5 has a higher ductility factor of 0.58, resulting in a considerable deflection. B3 and B4 beams are less ductile, with ductility values of 0.32 and 0.42, respectively.

### 4.3. Stress–Strain Behavior of Beams

**Figure 19:**
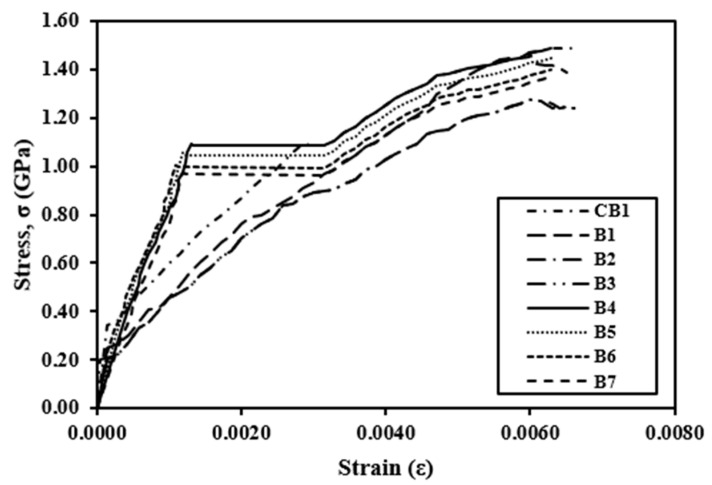
Stress–strain behavior of controlled and strengthened beams.

The stress–strain behavior of a control beam (CB1) and a strengthened beam at various stages of loading is depicted in Figure 19. At first, the stress distribution in concrete was linear. The distribution of concrete stress becomes nonlinear after a cracking load. The neutral axis shifts toward the compression zone of the cross-section as the load are increased. Until failure, the strain variation was almost linear. The proper anchorage of the CFRP sheet to the supports is responsible for the linear variation in strain. The proper anchorage prevented the CFRP sheet from debonding from the concrete.

### 4.4. Modes of Failures and Crack Patterns

#### 4.4.1. Different Types of Failures

**Figure 20:**
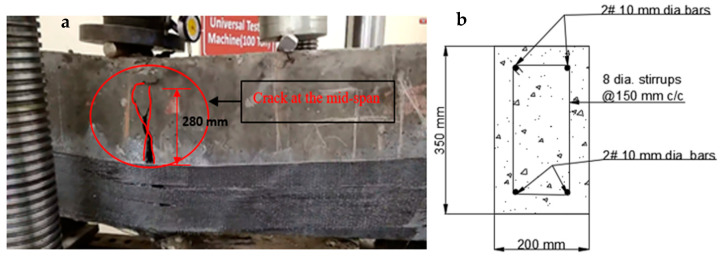
(**a**,**b**) Failure of the strengthened beam in the vertical direction.

**Figure 21:**
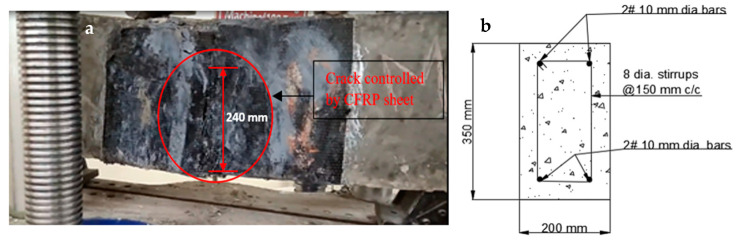
(**a**,**b**) Crack controlled in the strengthened beam.

**Figure 22:**
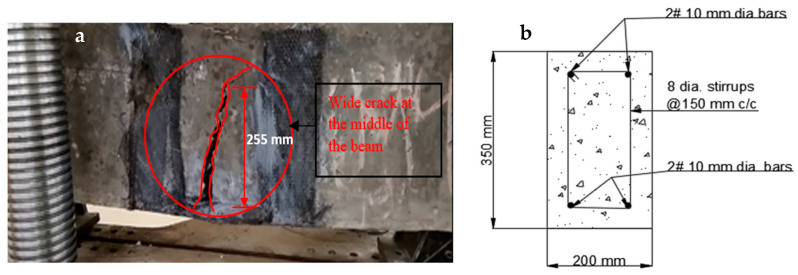
(**a**,**b**) Crack induced in a beam at the center.

The visual inspection was used to identify the failure modes of modified beams. Flexural failure induced by CFRP sheet debonding, steel rupture, and CFRP rupture were all identified [20,37]. On the beams in Series A, flexural failures with steel and CFRP ruptures were reported. The tension face flexural cracks were identified beneath the load areas. As the load increased, the bending cracks started to expand vertically upward, as seen in Figure 20a,b. These cracks were not widened because U-shaped CFRP sheets were provided. The B1 and B2 beams failed as a result of the steel rupture, while the CFRP sheets retained ductile. As flexural cracks intimated in B3 and B4 beams, the concrete was completely crushed at the soffit, and the CFRP sheet was broken in the center of the tension area of the beam, as shown in the figure. The B6 beam in Series B flexed due to concrete crushing, and the CFRP sheet was broken just in the middle of the beam. Like the B6 beam, the whole hogging moment region was U-wrapped, enabling crack visualization impossible. Concrete fracture and a damaged CFRP sheet in the middle of the tension side of the beam induced the beam to fail, as seen in Figure 21a,b. A flexural crack in the B5 beam was reported near the middle of the soffit. The flexural cracks began to expand vertically upward as the load progressed [37,72,73], and the beam failed to owe to a steel rupture as seen in Figure 22a,b, with the CFRP sheets. 

#### 4.4.2. Crack Patterns

The crack patterns are in one portion, and this part pertains to the flexural bending-induced crack at the mid-span of the beam. The amount of external FRP reinforcement and the specified loading conditions determine the manner of failure. The strengthened beams had a different crack propagation and crack pattern than the regulated beam. The strengthened beams had fewer cracks that were smaller in breadth, whereas the controlled beam had more cracks that were wider in width. It specifies that the cracks were restrained by the CFRP sheet [37,38,74]. In comparison to other retrofitted beams, the cracks in beams B1 and B4 are far fewer and smaller in size.

All CFRP-strengthened beams have much higher ultimate load capacities than the control beam. The surface treatment and CFRP design affect how much they are enhanced. This study shows that, in comparison to the full-length wrap and strip U-wrap of CFRP sheets under the loading regions, the full-length U-wrap system along the span of the beam is suitable for flexural strengthening, and the strip U-wrap system, which is placed alternately with the internal stirrups, is best for shear strengthening. Due to the externally bonded CFRP layer, the reinforced beams displayed high deflection values under ultimate and failure loads. The beams are therefore significantly more ductile and yield more than the control ones. Concrete crushing, rupturing, and debonding of the CFRP are the three primary failure mechanisms of the strengthened beams.

## 5. Conclusions

This study looked into the effectiveness of various flexural strengthening CFRP sheet techniques for retrofitting preloaded RC beams. The following conclusions were obtained:

- When CFRP sheets are used to reinforce preloaded damaged RC beams, functional stability is lost while the maximum load of the damaged beams is increased beyond that of the control beam. The CFRP sheet strengthening technique was more significant than the overall number applied to enhance the strengthening of damaged beams, as demonstrated in the Series A and Series B beams.
- Wrapping CFRP sheet across side faces of RC beams with tension face bonding provided adequate stiffness and bearing of the ultimate load to a significant extent, as seen in B3, B4, and B7 beams while wrapping the hogging moment zone of the beams with the approaches discussed effectively restored the beams’ strength.
- The U-shaped CFRP sheet anchors the tension facing CFRP and inhibits it from debonding. In preloaded beams, the use of a U-shaped CFRP sheet is advantageous. In the B1, B2, and B5 beams, the stiffness of retrofitted CFRP sheets was enhanced when contrasted to the stiffness of the control beam.
- At first, the stress distribution in concrete was linear. The distribution of concrete stress becomes nonlinear after a cracking load. The proper anchorage of the CFRP sheet to the supports is responsible for the linear variation in strain. The proper anchorage prevented the CFRP sheet from debonding from the concrete.
- The deformation of the retrofitted beam is reduced to a point below the control beam when the CFRP sheet is utilized for retrofitting. CFRP sheet retrofitting beams have more reduced failure widths than the control beams. The study reveals that flexural failure is the most common failure mode, including concrete crushing and steel rupture, which improve retrofitting effectiveness.

## Figures and Tables

**Table 1:** Characteristics of ordinary Portland cement 43 Grade.

Sr. No.	Property	Experimental Values	Code Provision IS: 8112:2013
1	Blaine’s Fineness (cm^2^/gm)	-	2250
2	Normal Consistency (% Age of Cement by Weight)	29	-
3	Setting Time (min)		
	a. Initial Setting Time (min)	125	>30
	b. Final Setting Time (min)	355	<600
4	Compressive Strength (MPa)		
	a. 3 Days	26	23
	b. 7 Days	39	33
	c. 28 Days	47	43

**Table 6:** Physical and chemical properties of superplasticizer.

Properties	Value
Tensile Strength (MPa)	4000
Color	Black
Modulus of Elasticity (MPa)	230
Physical State	Liquid
Ph (concentrate)	≥6
Chemical Base	Modified Polycarboxylate
Odour	Slight/faint
Water solubility	Soluble

## Data Availability

No data were used to support this study.
